# Importance of the association of molecular and immunological diagnosis in immunocompetent patient with *Histoplasma capsulatum* and *Cryptoccocus neoformans* infection: a case report

**DOI:** 10.1186/1678-9199-20-36

**Published:** 2014-08-26

**Authors:** Kátia Cristina Dantas, Roseli Santos de Freitas, Roberta Scholz Pinto Garcia, Marcos Vinícius da Silva, Edna Cleide Mendes Muricy, Valdelene Sayuri Kohara, Adriana Pardini Vicentini

**Affiliations:** 1 Department of Pathology, Medical School, University of São Paulo, São Paulo, São Paulo State, Brazil; 2 Laboratory of Medical Mycology – LIM 53/HCFMUSP and Institute of Tropical Medicine, University of São Paulo, São Paulo, São Paulo State, Brazil; 3 Emílio Ribas Infectious Diseases Institute, São Paulo, São Paulo State, Brazil; 4 Immunology Center, Adolfo Lutz Institute, Av. Dr. Arnaldo, 351, 11° andar, sala 1117, São Paulo, São Paulo State 01246-902, Brazil

**Keywords:** Immunocompetent individuals, Nested PCR, Immunoblotting, Fungal infections, Blood fluid

## Abstract

This case reports an immunocompetent 29-year-old woman with suspected pneumonia, suggestive of fungal infection. Immunoblotting analysis reactivity against *Histoplasma capsulatum* and *Paracoccidioides brasiliensis* were observed. Nested-PCR in blood employing species-specific primers was positive for *H. capsulatum* and *Cryptococcus neoformans.* The evaluation of paucisymptomatic patients with positive results for *H. capsulatum* and *C. neoformans* could be relevant for the prevention as well as the possible evaluation of the reactivated quiescent foci. In conclusion, the associated methodology may have contributed to the monitoring endogenous reactivation of these diseases.

## Background

Fungal infection of the respiratory tract can take several forms, the most common of which is pneumonia [1]. However, the number of etiologic agents attacking immunocompetent individuals and causing significant infection is limited. The most common of these are *Aspergillus* spp., *Pneumocystis jirovecii*, *Cryptococcus* spp., *Histoplasma capsulatum, Scedosporium* spp., *Fusarium* spp. and *Candida* spp. [1].

Histoplasmosis (HP) is a systemic disease caused by the thermally dimorphic fungus *Histoplasma capsulatum*. HP is a cosmopolitan mycosis with areas of particularly high endemicity [2]. In Brazil, endemic areas are located in the midwestern and southeastern regions of the country [3]. Humans infected by *H. capsulatum* experience an unapparent or mild disease and usually develop a positive reaction to a histoplasmin skin test within two weeks after exposure [2,4]. The clinical spectrum ranges from asymptomatic, self-limited illness to a life-threatening progressive disseminated disease [2].

*Cryptococcus* spp. are widespread fungi in many parts of the world, and are responsible for cryptococcosis, the most common opportunistic infection in patients with AIDS [5,6]. The acute form of the disease is more common in immunodeficient patients, such as those infected with HIV, those treated with corticosteroids, and those with other types of immunodeficiency (related to tuberculosis, diabetes mellitus, hematologic cancers, or chemotherapy). A chronic form characterized by discrete mild symptoms or no symptoms is observed in immunocompetent individuals [6]. In Brazil, the major cases of cryptococcosis in regions south, southwest and central-east occurred in patients with AIDS. In addition, findings from the northern and northeastern regions show that this infection may also be associated with outbreaks [5].

Concomitant cryptococcosis and histoplasmosis are rarely reported in the literature. Aronis *et al.*[7] reported concomitant disseminated histoplasmosis and cryptococcosis in a 20-years-old Brazilian woman with AIDS. Furthermore, the authors reported cases described in the medical literature showing, from January 1970 to December 2010, only five cases of coinfection by *H. capsulatum* and *C. neoformans* in patients in Europe and South and North America [7].

The infection occurs after inhalation of the fungal propagules by the host [4]. Infections often follow human disruption of the soil during spelunking, farming, cleaning attics, bridges or barns, construction and tearing down old structures laden with bat and/or bird guano; all of these activities tend to aerosolize the fungal propagules. The risk of infection as well as the spectrum of clinical manifestations depend mainly on the magnitude of exposure, professional occupation and activity duration. The manifestations of mycoses were influenced by genetic and environmental conditions such as the amount of the fungal propagules in the soil, the immune status of the host and the virulence of the infecting strain [8]. Epidemiology of fungal infections is important in immunocompetent individuals, especially when the available diagnostic tools do not show conclusive results [9,10].

Definitive diagnosis is still based on the isolation and identification of the etiological agent, by growth of the fungi from sputum, blood, tissue biopsy or biological specimens [8,9,11]. If neither cultural nor morphological proof of infection is available, other approaches must be used [8,9,11]. In contrast to other endemic mycoses, serologic assays for detection of specific host antibody responses and/or antigen detection often provide this additional information for the diagnosis of histoplasmosis and cryptococcosis [4,6,9,12].

Because of their convenience, availability, utility and accuracy, the most widely accepted immunological assays are immunodiffusion, complement fixation and latex aggluttination [5]. However, these methods have limitations associated with false positivity, mainly in patients with the disseminated form of disease. False positive results occur most frequently in the presence of cross-reactive epitopes shared with other fungal pathogens like *Paracoccidioides brasiliensis*, *Coccidioides immitis* and *Aspergillus* sp [8,9,13]. In addition, Yeo and Wong [13], in a review article, showed that DNA-based diagnostic tests have the potential to reduce the time for laboratory identification of pathogens that are slow growing or difficult to culture. Guedes *et al.*[12] employed the PCR method for the detection and identification of *H. capsulatum*. The PCR assay was developed with specific primer pairs whose sequences were based on the *H. capsulatum* M-protein gene sequence, a test proven sensitive and specific for the identification of typical and atypical isolates of this fungal species. The primer pairs were specific for *Cryptococcus* spp. whose sequence was based on the 18S rDNA region [14].

We report herein a case of histoplasmosis and cryptococosis infection in a young HIV-negative woman. She had visited the countryside of Bahia, in northeastern Brazil. Our goal was to demonstrate the importance of the association of molecular and immunological diagnoses in a blood sample from an immunocompetent patient with suspected fungal infection.

## Case presentation

A immunocompetent 29-years-old woman patient, who lives in São Paulo, was referred to the Emilio Ribas Institute in January 2008 with suspected pneumonia. At that time, the patient was not taking any medication and had no history of systemic disease. She did not report previous blood transfusions or surgeries. The patient reported a history of traveling to a rural area in Bahia state, Brazil. Importantly, she related that she had direct contact with chicken excreta while cleaning a coop and had taken inadequate precautions.

Approximately 20 days after returning to São Paulo, the patient presented intermittent fever, productive cough, chest pain, dyspnea, and myalgia for three months. A general clinical examination revealed a patient with normal skin color, afebrile, hydrated, non-cyanotic and non-edematous; no lymphadenopathy or hepatosplenomegaly was observed. The abdomen was soft and non-rigid with normal bowel sounds. Cardiac auscultation revealed a regular rate and rhythm, and normal breath sounds. Antibodies against HIV, viral hepatitis, leishmaniasis, Chagas’ disease, malaria, paracoccidioidomycosis, histoplasmosis and aspergillosis, as well as skin tests for paracoccidioidin and histoplasmin were negative. Serum cryptococcal antigen latex agglutination test was negative. Direct sputum examination was negative in three samples for fungus and acid-alcohol resistant bacillus. The isolation from serial blood samples collected and inoculated under sterile conditions on Sabouraud-dextrose brain-heart infusion (Difco Laboratories, USA), and tryptone soya broth (Oxoid, UK), incubated at 30°C and at 35°C were all negative. Radiological images proved to be inconclusive, with no pathological changes.

During the clinical evaluation, the patient had spontaneous resolution of the clinical picture; therefore, it was not necessary to request additional tests such as CT or echocardiogram. The epidemiological history was compatible between histoplasmosis and cryptococcosis. Double immunodiffusion and immunoblotting assays were performed according to Freitas *et al.*[9], whereas the serum cryptococcal antigen latex agglutination test was performed using the IMMY kit (Immuno-Mycologics, USA), according to the manufacturer’s instructions. Double immunodiffusion (DI) produced no detectable reactivity of the serum samples against *H. capsulatum* and *P. brasiliensis* antigens. By immunoblotting there was reactivity with the H and M fractions of *H. capsulatum* as well as gp43 of *P. brasiliensis*. DNA was extracted by means of a kit (QIAamp Kit Blood Mini genomico; QIAgen, USA). The reaction conditions and the primers were chosen according to previous studies [14-18]. Negative (water) controls were tested simultaneously.

All reactions were accompanied by either a negative control (no added DNA), or a positive control (strains references for *H. capsulatum* – 200, *C. neoformans* – ATCC 24067, and *P. brasiliensis* – 18 and B-339) and negative group control (seven healthy individuals). Blood and serum samples from the patient were evaluated molecularly over 12 months. During this period, we detected DNA for *C. neoformans* for eight months, versus six months for that of *H. capsulatum*. Nested PCR reactions were processed and analyzed three times. The presence of amplified DNA was confirmed by a nested PCR using a target sequence of GenBank accession number J04038.1 of the human glyceraldehydes-3-phosphate dehydrogenase (GADPH) as elsewhere described [19]. The nested-PCR in blood and serum samples, using species-specific primers HC18S, HC100, PBGP43 and CN18S were positive for *H. capsulatum* and *C. neoformans*, but negative for *P. brasiliensis*, showing the possible infection by *H. capsulatum* observed in immunoblotting [14-18].

The PCR product of the conserved region 5.8S rDNA-ITS was obtained under the conditions described by Fujita *et al.*[20]. Purification of this product was performed with the PureLink kit (Invitrogen, Life Technologies, USA). From this purified product, sequencing was performed using MegaBACE 1000, a system for simultaneous DNA analysis with 96 capillaries from GE Healthcare, CA, USA). The sequencing reactions were performed according to the protocol for the Mega BACE 1000, using DYEnamic ET Dye Terminator kit (with Thermo Sequence II DNA Polymerase, GE Healthcare, USA). All sequences were analyzed by means of the software Sequence Analyzer, using the Base Caller Cimarron 3.12. The sequences were identified using the BLAST program (http://www.ncbi.nlm.nih.gov/BLAST). Furthermore, multiple alignments were assembled using ClustalW (http://www.ebi.ac.uk). The sequences resulting from this study were made available for public databases. The results of sequencing showed that all strains were *C. neoformans* and exhibited 98% similarity with the neoformans variety. The sequencing of strains confirmed the results of tests that involved Nested PCR.

### Discussion

In this study, we show the relevance of laboratory diagnosis in the clinical setting of an immunocompetent patient. According to the literature, the clinical manifestation by fungal infection can range from asymptomatic infection to dissemination [4,21-23]. The patient of this study showed the initial clinical suspicion of pneumonia. However, the results of radiological images, according to Wheat and Kauffman [2], are commonly negative in immunocompetent patients. In relation to the treatment, the patient did not require therapy with spontaneous resolution, confirming the description by Wheat *et al.*[24] and Bicanic and Harrison [25] in immunocompetent patients.

Although this case was reported in São Paulo, we have strong evidence that the patient had become infected in the Brazilian state of Bahia. Thus, we believe in the existence of microfoci for both *H. capsulatum* and for *C. neoformans*, as found in a rural chicken coop or in soil. Despite the description of cases in northeastern Brazil, this region is not considered endemic [26-29]. According to Guimarães *et al.*[3] the northeast presents a histoplasmosis prevalence of 2-29%. In Ceará, the incidence of histoplasmosis has more than doubled among patients with AIDS in the past decade [26,30,31]. Fortaleza *et al*. [10] reported one case of an immunocompetent patient with histoplasmosis that developed a severe form of the disease, presumably due to the intensity of exposure and delayed diagnosis.

Cryptococcosis and histoplasmosis are the most common fungal life-threatening infections in AIDS patients. However, coinfection with these pathogens is rarely described in the literature [7]. In this study, we show a case of an immunocompetent patient with strong evidence of coinfection by *H. capsulatum* and *C. neoformans*. However, due to the association of serological and more sensitive molecular methods, their detection was possible from the early phase of fungal infection. The mycological diagnoses from serial blood samples were negative, according to Wheat [32], in immunocompetent individuals. This demonstrated that the culture has low sensitivity to isolated fungi [4,11,32].

Serological evaluation is central to the diagnosis of some acute, recent or chronic infectious diseases. In histoplasmosis infections, the detection of patients’ antibody responses offers a more rapid alternative to microbiological means of diagnosis, and the detection of host anti-*H. capsulatum* antibodies by immunodiffusion (ID). The scarcity of the fungus in clinical specimens and a negative serology result due to anergy or the low sensitivity of the assays are common problems [9].

**Figure 1:**
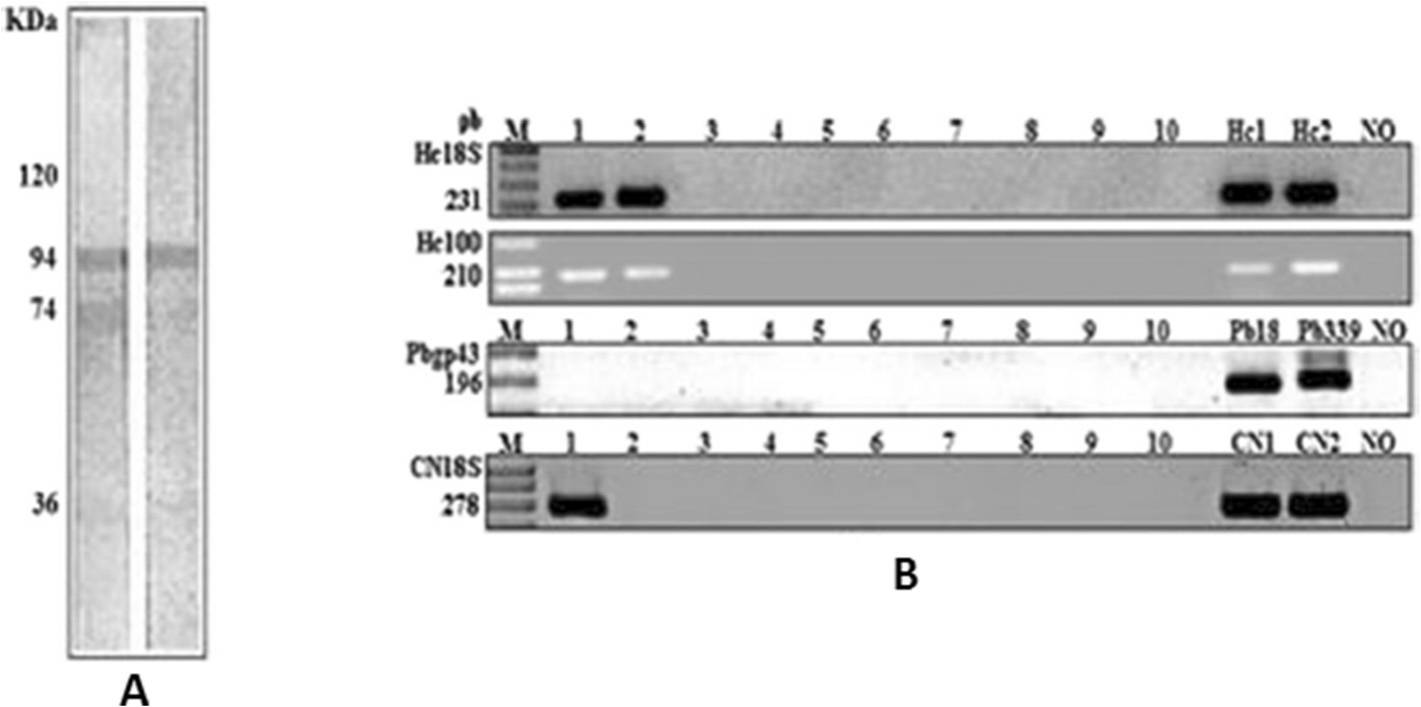
**Immunoblotting and PCR assays. (A)**: Immunoreactivity of serum patient against H and M fractions of *H. capsulatum*. **(B)**: Lane M. DNA molecular weight marker 1kb DNA ladder; Lane 1. patient serum collected in February 2008; Lane 2.- patient serum collected in June 2008; Lanes 3 to 10. negative individual sera samples, Lane Hc1. *H. capsulatum* strain (immunodeficiency); Lane Hc2. *H. capsulatum* 200 strain; Lane Pb 18. *P. brasiliensis* strain; Lane Pb 339 *P. brasiliensis* 339, Lane CN1 e CN2. *C. neoformans* strains (ATCC 24067) and Lane NO. negative control.

Katzenstein [33] studied specificity of skin tests in deep fungal infection, showing that the response to skin testing is closely related to the concentration of antigen employed. In this paper, the author confirms the anergic state, since the reaction of delayed-type hypersensitivity showed no response. Infection by H. capsulatum was later confirmed at autopsy [33]. The reaction of nested PCR was positive for *H. capsulatum* and *C. neoformans*, and negative for *P. brasiliensis*, showing the possible infection by *H. capsulatum* observed in immunoblotting (Figure 1A).

The recognition of the 43 kDa glycoprotein of *P. brasiliensis* in immunoblotting may be due to cross reactivity with similar epitope of these two pathogens [9]. In this report, we used nested PCR for the diagnosis of fungal infection and confirmed that this assay is specific for *H. capsulatum* and *C. neoformans* and could detect 1 cell/mL. Therefore, this technique was considered another useful method for the diagnosis of histoplasmosis and cryptococcosis. The primer sequences used in this study were based on the small-subunit (18S) rDNA gene and specific 100 kDa protein of *H. capsulatum*[34]. They did not show any false-positive results and were able to detect fungal DNA in serum and blood samples [14,16,18]. The positive result for *Cryptococcus* spp., in which fungal DNA was detected in serum and blood samples suggested a concomitant infection, principally by epidemiological similarity (Figure 1B).

The result of these studies is that PCR can be a useful additional tool for diagnosis of *H. capsulatum* and *C. neorformans*, especially in non-endemic regions, to complement antibody detection and serological methods. The monitoring of asymptomatic patients with confirmed infection by *H. capsulatum* and *C. neoformans* may also have relevance in the prevention and monitoring of possible reactivation of quiescent foci, common in systemic mycoses, and thus contribute to the pathogenesis and epidemiological knowledge of these diseases.

The nested PCR tested negative for detection of DNA fragment specific for *P. brasiliensis*, which seems to corroborate the hypothesis of cross-reactivity, as this technique is more specific than immunoblotting. The patient's serum presented the fragment of the 18S region, conserved for fungi, as well as the specific primer region of the gene that encodes a protein of 100 kDa (fraction M of *H. capsulatum*). Figure 1 displays the PCR results from the nucleic acids extracted from the blood and serum. GAPDH nested PCR products obtained from this patient positively confirmed that the samples were from human material.

In view of the seriousness of fungal infection, and the scarcity of data on improvement of procedures in the detection and identification of fungi, this study seems to present clinically relevant and actionable results. We believe that testing of biological fluids (blood and serum) with primer sequences targeting rDNA and ITS regions for the detection of *H. capsulatum* and *C. neoformans* will improve diagnostic procedures and monitoring of these infections. It is necessary to point out that in clinical practice, collection of biological samples after the onset of severe symptoms and failure to detect small concentrations of a fungal antigen prevent early laboratory diagnosis of histoplasmosis and cryptococcosis, timely initiation of therapy, and accurate prognosis of the disease. We expect that a molecular diagnostic test diagnosis is a fast and reliable means of improving the management of cryptococcosis and histoplasmosis.

## Conclusion

The present case reinforces the idea that other more sensitive and specific diagnostic methods, such as the immunosorbent and molecular biology methods, are necessary to confirm a diagnosis, especially when these patients have generated a strong clinical suspicion. Therefore, in the presence of negative serologic reactions, the molecular biology diagnostic tools are important for confirming the fungal infection, thereby allowing diagnosis and successful therapy and prognosis.

## Consent

Informed consent was obtained from the patient for publication of this report. The research project was submitted for analysis and approved by the Ethics Committee for Research on Human Subjects of the Adolfo Lutz Institute (CEPIAL, n. 21/09), Ethics Committee for Research on Human Subjects of the Emílio Ribas Infectious Diseases Institute (CEP, n. 296/11) in accordance with Resolution 196/96 of the Brazilian National Health Council (Conselho Nacional de Saúde do Brasil).

## Competing interests

The authors declare that there are no competing interests.

## Authors’ contributions

MVC described the case. KCD, RSF, RSPG, ECMM and VSK conducted laboratory evaluations (mycological, immunological and molecular assays). KCD and RSF drafted the first version of the article. KCD, RSF, MVS and APV made a critical revision of the text. All authors read and approved the final manuscript.
